# Renal and Cardiovascular Toxicities by New Systemic Treatments for Prostate Cancer

**DOI:** 10.3390/cancers12071750

**Published:** 2020-07-01

**Authors:** Giuseppe Saltalamacchia, Mara Frascaroli, Antonio Bernardo, Erica Quaquarini

**Affiliations:** 1 Operative Unit of Medical Oncology, IRCCS Istituti Clinici Scientifici Maugeri, 27100 Pavia, Italy; giuseppe.saltalamacchia@icsmaugeri.it (G.S.); antonio.bernardo@icsmaugeri.it (A.B.); 2 Department of Internal Medicine and Therapeutics, University of Pavia, 27100 Pavia, Italy; 3 Operative Unit of Translational Oncology, IRCCS Istituti Clinici Scientifici Maugeri, 27100 Pavia, Italy; mara.frascaroli@icsmaugeri.it; 4 Experimental Medicine School, University of Pavia, 27100 Pavia, Italy

**Keywords:** abiraterone, apalutamide, cabazitaxel, darolutamide, enzalutamide, radium-223

## Abstract

Prostate cancer (PC) is the most common male cancer in Western Countries. In recent years, the treatment of relapsed or metastatic disease had benefited by the introduction of a variety of new different drugs. In consideration of the relative long survival of PC patients, side effects of these drugs must be considered and monitored. In this review, we analyzed the newly developed therapies for PC treatment, describing the mechanism of action, the metabolism and latest clinical trials that led to the approval of these drugs in clinical practice. We then evaluated the cardiovascular and renal side effects from pivotal phase III and II studies and meta-analyses. Cardiovascular side effects are the most frequent, in particular hypertension, while renal toxicity is rarer and not well described in literature. Therefore, there is a need to better define the effects of these therapies, in order to personalize patient treatment on the basis of their comorbidities and preferences, in addition to their symptoms and disease load.

## 1. Introduction

Prostate cancer (PC) is the most frequent malignancy in the male population of the Western world. Its incidence increased until 2003, with a subsequent reduction (−1.4%/year), and it now accounts for 20% of cancers diagnosed in male patients older than 50 [1]. The rising incidence is directly associated with the improvement of the diagnostic techniques, such as prostate specific antigen (PSA) dosage that has profoundly modified the epidemiology of this tumor [2]. PC is the third predicted cause of cancer death in European men, with 78,800 deaths in 2020 and a rate of 10.0/100,000 [3]. 

Localized PC is in generally treated with surgical intervention, radiotherapy, hormonal therapy or just observation, while the treatment of localized high-risk or metastatic disease (endocrine sensitive or castration resistant) may be challenging. Androgen deprivation therapy (ADT) has represented a cornerstone for treating locally advanced and metastatic disease. Until 2004, progression on ADT for metastatic castration-resistant prostate cancer (mCRPC) was treated with the addition of secondary hormonal manipulation, including antiandrogens such as bicalutamide and nilutamide, ketoconazole, or corticosteroids [4]. Mitoxantrone, the first cytotoxic chemotherapy approved for mCRPC by the US Food and Drug Administration (FDA), was approved on the basis of improved palliative responses in pain-related measures, despite no survival benefit [5]. Docetaxel, a microtubule inhibitor, represented the first systemic therapy to demonstrate survival benefit in mCRPC in two prospective phase III trials in 2004, thus becoming a standard of care in this setting [6,7]. In recent years, different drugs for the treatment of localized high-risk or metastatic disease have been approved and several clinical trials are still ongoing. The therapeutic armament has become wide: abiraterone, a CYP17 inhibitor; enzalutamide, an androgen receptor antagonist; apalutamide, a non-steroidal anti-androgen (NSAA); cabazitaxel, a new taxane; sipuleucel-T, an autologous cellular immunotherapy; and radium-223, an alpha-emitter [8].

New treatment-related toxicities are emerging with the introduction of new drugs; renal and cardiovascular side effects are particularly interesting since PC often follows a prolonged disease course that generally affects an elderly male population with concurrent medical conditions. Indeed, in multiple studies evaluating the cause of death in men with PC, non-cancer-related events (in particular ischemic cardiac events) have emerged to be the most common ones [9,10].

Cardiovascular toxicities might occur during or after the completion of an anticancer treatment. They are more common in patients over 50 years old, males and in the presence of genetic and environmental factors, a family risk of coronary heart disease or heart failure and comorbidities such as diabetes and dyslipidemia [11].

ADT is associated with numerous metabolic alterations, overlapping with the metabolic syndrome (weight gain, visceral adiposity, insulin resistance, increased arterial stiffness, and less favorable lipid profiles) that increase the risk of cardiovascular death [12]. Several studies had described the treatment-related cardiovascular risk associated with the use of ADT in men with PC [13,14]. However, a subsequent meta-analysis did not find any association between ADT and cardiovascular disease but it underlined a lower risk of disease-related and all-cause mortality during ADT [15]. In addition, ADT can increase the risk of kidney injury due to metabolic alterations and by lowering testosterone to castration levels, antagonizing the vasodilating effects of testosterone on renal vessels [16,17]. Several studies described a potential kidney injury during bicalutamide treatment. In fact, the drug may provoke renal mesangial damage and fibrosis via different mechanisms: androgen deprivation, upregulating of TNF-α→NF-κB→caspase-3 death pathway, and downregulating the PI3K-Akt survival pathway and PDGF-fibronectin-collagen IV fibrogenic pathway [18,19]. Furthermore, patients who received ADT as part of a combination therapy had the highest risk of acute kidney injury, suggesting that ADT may potentiate the nephrotoxicity of other agents. In addition, age is a major risk factor for kidney disease. The decline in renal function is common in the elderly population; by the age of 70 years, renal function may decline by up to 40%. Other identified risk factors include hypertension and cardiac diseases so that cardiovascular and renal toxicities may be strictly linked.

In literature, the potential cardiovascular and renal adverse events of new PC treatments have not been systematically summarized yet. In this review, we analyzed the new therapies for PC treatment approved in the last 10 years, describing the mechanism of action, the metabolism and latest clinical trials that led to the approval of these drugs in clinical practice. We then focused on their cardiovascular and renal side effects, taking in consideration articles published in the English language of phase III or phase II randomized controlled trials and meta-analyses. The cardiovascular toxicities we considered included hypertension, atrial fibrillation, ischemic heart disease, myocardial infarction, arrhythmias, and cardiac failure; the renal sides effects included hematuria, renal failure, and urinary retention.

## 2. New Hormonal Treatments

### 2.1. Apalutamide

#### 2.1.1. Mechanism of Action, Metabolism and Clinical Use 

Apalutamide is a non-steroidal androgen receptor (AR) inhibitor, belonging to the new-generation class of AR antagonist. The drug binds directly to the ligand-binding domain of AR, preventing AR translocation, DNA binding, and AR-mediated transcription [20]. Apalutamide is rapidly adsorbed following oral administration and it reaches the time of maximum concentration (t-max) in two hours. It is metabolized to N-desmethyl apalutamide (its active metabolite) by CYP2C8 (cytochrome P450) and CYP3A4. Seventy days after the radiolabelled dose, 65% of the drug was observed in urine and 24% recovered in feces [21]. Apalutamide pharmacokinetics is mostly mediated by CYP2C8 and CYP3A4 and several interactions with other drugs, which are inducers of these cytochromes, have been described. The co-administration of strong CYP2C8 inhibitors, such as gemfibrozil, has been reported to determine an apalutamide area under the curve (AUC) increasing of about 68% [22]. In contrast, a single dose of CYP3A4 inhibitor, such as itraconazolo, has no effect on apalutamide clearence. Apalutamide also has an inducer role on CYP3A4: the concomitant administration of treatments metabolized by CYP3A4, such as midazolam, omeprazole, warfarin and rosurvastatin, determines a reduction of the bioavailability of these drugs. The drug was approved by the FDA in 2019 and by the European Medicines Agency (EMA) in 2019 for the treatment of men with non-metastatic castration-resistant PC (nmCRPC) at high risk of developing metastatic disease and with metastatic hormone-sensitive PC (mHSPC) in combination with ADT [23].

#### 2.1.2. Clinical Trials

The Spartan trial is a phase III study enrolling 1207 high-risk men affected by nmCRPC and randomized 2:1 to receive apalutamide versus placebo [24]. High-risk features were defined as having a PSA doubling time of 10 months or less. The primary objective of the trial was the metastasis-free survival interval and it was met since it resulted to be 40.5 months in the apalutamide group as compared with 16.2 months in the placebo group (HR = 0.28; *p* < 0.001).

The ongoing phase III ATLAS study is enrolling patients with high-risk nmHSPC (Gleason score of ≥8 and ≥cT2c or Gleason score of ≥7 and PSA ≥ 20 ng/mL and ≥cT2c) affected by localized or locally advanced PC receiving primary radiation therapy [25]. Patients are randomized 1:1 to receive apalutamide versus bicalutamide in neoadjuvant/concurrent to radiotherapy phase (cycles 1–4) and apalutamide versus placebo in adjuvant phase (cycles 5–30). The primary endpoint of the study is metastasis-free survival.

The Titan trial is a phase III trial in which 1052 patients with mHSPC were randomized 1:1 to receive apalutamide versus placebo in addition to ADT [26]. The co-primary endpoints were radiographic progression-free survival (PFS) and overall survival (OS). The results favored the experimental arm since the percentage of patients with radiographic PFS at 24 months was 68.2% in the apalutamide group and 47.5% in the placebo group (HR = 0.48; *p* < 0.001). OS at 24 months was longer in the experimental arm than in placebo one, being 82.4% versus 73.5% in the apalutamide and in the placebo group, respectively (HR = 0.67; *p* = 0.005). 

#### 2.1.3. Renal and Cardiac Toxicities

In the Spartan trial, 199 patients (24.8%) treated with apalutamide developed hypertension of all grades, whereas 115 (14.3%) of grade 3–4. Cardiac toxicities lead, in a small number of cases, to treatment interruption and, in particular, hypertension (1.2% of cases) and atrial fibrillation (0.7%). 

In the Titan trial, 93 (17.7%) patients treated with apalutamide developed hypertension of all grades, accounting grade 3–4 events for 44 (8.4%) cases. Ischemic heart disease occurred in 23 (4.4%) patients and caused death in two patients. 

Belderbos et al. published an open label, multicentre, phase Ib study, investigating the effect of apalutamide on ventricular repolarization in men with CRPC [27]. A total of 55 patients were enrolled. Electrocardiogram (ECG) measurements were performed before the start of the treatment, and on days 1–57 (cycle 3 day 1). Apalutamide pharmacokinetics was evaluated at days 1–57 at matched points of the ECG collection. The QT interval was corrected using Friedericia correction (QTcF). Grade 1–2 cardiac toxicities were reported in 73% of patients due to the presence of a maximum corrected QT interval of 12.4 ms (normal value 0.33–0.45) but no patients stopped the experimental treatment due to prolonged QT interval. In conclusion, the effect of apalutamide on QT was modest and not acting negatively on ventricular repolarization.

The impact of apalutamide administration in case of severe renal impairment is not established due to a lack of data. At the same time, clinical trials do not describe significant renal toxicities during apalutamide treatment. In the Spartan trial, renal toxicities lead to treatment interruption in a small number of cases (hematuria: 1.1%, and acute kidney injury: 0.4%). In the Titan trial, urinary retention occurred in 2.5% of patients, with grade 3–4 events in <1% of cases.

### 2.2. Darolutamide

#### 2.2.1. Mechanism of Action, Metabolism and Clinical Use

Darolutamide is a non-steroidal AR antagonist with a different molecular structure compared to other AR antagonists. The drug is formed by two pharmacologically active substances (S,R-darolutamide and S,S-darolutamide), which interconvert through the pharmacologically active major metabolite keto-darolutamide [28,29]. The drug is mainly excreted in urine (63.4%). Although preclinical data indicate that darolutamide exposure may be affected by drugs that are strong CYP3A4 inducers or inhibitors, no significant associations between the use of concomitant medications and the pharmacokinetics variability of darolutamide have been reported [30]. Darolutamide was approved by the FDA in 2019 and by the EMA in 2020 for the treatment of patients with nmCRPC at high risk of developing metastasis. [31].

#### 2.2.2. Clinical Trials

The ARADES trial, a phase I/II study, enrolled men with progressive mCRPC, who had castrate concentrations of testosterone and an Eastern Cooperative Oncology Group (ECOG) score of 0–1 [32]. In the first phase of the study, 24 patients received an increasing progressive dose of darolutamide (from 200 mg/daily to 1800 mg daily). In the second phase, 110 men were added and were randomly assigned to receive three different daily doses of darolutamide (200 mg, 400 mg, and 1400 mg). The primary endpoint of this phase was the proportion of patients with a PSA response (50% or greater decrease in serum PSA) at 12 weeks. The results show that 11 (29%) patients in the 200 mg group, 13 (33%) in the 400 mg group, and 11 (33%) in the 1400 mg group had a PSA response at 12 weeks.

Furthermore, two phase III clinical studies, the ARAMIS and ARASENS trials, were performed [33,34]. The first one examined the safety and efficacy of darolutamide in 1509 men with nmCRPC. Patients were randomized 2:1 to receive darolutamide plus ADT versus placebo plus ADT. The primary endpoint of the trial was metastasis-free survival and it was met since median metastasis-free survival was 40.4 months versus 18.4 months in the experimental and placebo arm, respectively (HR = 0.41; *p* < 0.001). The second study randomized in a 1:1 ratio 1300 patients with mHSPC to receive darolutamide plus ADT plus docetaxel versus placebo plus docetaxel plus ADT. The primary endpoint of the trial is OS and the results are still not mature. 

#### 2.2.3. Renal and Cardiac Toxicity

Few cardiac events are described during darolutamide treatment. In the ARADES study, one (<1%) patient developed grade 1 hypertension, seven (6%) grade 2 and one (<1%) grade 3. In the ARAMIS trial, a total of 63 (6.6%) patients developed hypertension, including 30 (3.1%) cases of grade 3–4; coronary disorders occurred in 31 (3.2%) patients, including 16 (1.7%) of grade 3–4, and heart failure in 18 (1.9%) patients, including five (0.5%) of grade 3–4. 

With regards to renal toxicity, it was not described in the ARADES trial, whereas, in the ARAMIS study, urinary retention was observed in 33 (3.5%) men, 15 (1.6%) of grade 3–4. However, considering the metabolism of the drug, patients with severe renal impairment who are not receiving hemodialysis have a higher exposure to darolutamide and a reduction dose is recommended; the effect of darolutamide in patients with end-stage renal disease has not been studied yet.

### 2.3. Abiraterone Acetate

#### 2.3.1. Mechanism of Action, Metabolism and Clinical Use

Abiraterone acetate, a prodrug of abiraterone, is a selective inhibitor of androgen biosynthesis that potently blocks cytochrome P450 c17 (CYP17), a critical enzyme in testosterone synthesis, thereby blocking androgen synthesis by the adrenal glands and testes and within the prostate tumor [35]. The metabolism of abiraterone is predominantly hepatic and it is excreted by feces. Abiraterone acetate is metabolized by the enzyme sulfotransferase 2A1 and by CYP3A4 [36]. A recent study evaluated the interactions between hormonal treatment (including abiraterone) and different medications in 45 patients affected by mCRPC. The most frequent medications were: lansoprazole (47%), oxycodone (27%), paracetamol (22%), statins (13%), codeine (13%), and warfarin (4%). It has been reported that, since lansoprazole is metabolized by CYP2C19 and CYP3A4, the cytochromes are both moderately inhibited by abiraterone, while oxycodone, codeine and paracetamol are metabolized by CYP2D6 that is strongly inhibited by abiraterone. The FDA and EMA initially approved abiraterone in 2011 as a treatment for mCRPC. Then, extensions of this indication were performed by the FDA in 2018 and the EMA in 2017, so now it is approved in combination with prednisone or prednisolone in patients with: 1) high-risk and newly diagnosed mHSPC in combination with ADT; 2) asymptomatic or mildly symptomatic patients with mCRPC after the failure of an ADT, for whom chemotherapy is not yet clinically indicated; 3) patients with mCRPC, whose disease has progressed during or after treatment with docetaxel [37].

#### 2.3.2. Clinical Trials

The phase III trial COU-AA 301 randomized in a 2:1 ratio 1195 patients with mCRPC to receive abiraterone acetate plus prednisone versus placebo, after a chemotherapy regimen with docetaxel [35]. The primary endpoint was OS, and it was met since patients in the abiraterone group had a longer survival than patients in the placebo one (14.8 months versus 10.9 months, respectively; HR = 0.65; *p* < 0.001). The COU-AA-302 is another phase III trial in which 1088 patients with asymptomatic or mildly symptomatic chemotherapy-naive mCRPC were randomized in a 1:1 ratio to receive prednisone 5 mg twice daily plus abiraterone acetate 1000 mg versus placebo [38]. The primary endpoint of the study was OS and it favored the experimental arm (34.7 months versus 30.3 months in the experimental and in placebo group, respectively; HR = 0.81; *p* = 0.0033).

Thanks to the results of the phase III STAMPEDE trial, abiraterone acetate plus ADT was approved for the treatment of patients with high-risk, treatment-naive, metastatic or non-metastatic PC [39]. In this study, 1917 patients were randomized 1:1 to receive ADT alone versus ADT plus abiraterone acetate and prednisolone. The primary endpoint was OS and, at a median follow-up of 40 months, it was met since the experimental arm had higher survival than the control one (184 deaths versus 262, respectively; HR = 0.63; *p* < 0.001). The recorded HR was 0.75 for non-metastatic patients and 0.61 for metastatic men. 

The phase III LATITUDE study randomized in a 1:1 ratio 1199 patients with newly diagnosed, metastatic, high-risk PC to receive abiraterone acetate plus prednisone and ADT versus placebo plus ADT [40]. High-risk features were defined as the presence of at least two of these three prognostic factors: a Gleason score ≥8, the presence of three or more lesions on a bone scan, or the presence of measurable visceral metastasis except lymph node metastasis. A primary endpoint was OS and, at the final analysis, abiraterone plus prednisone treatment had recorded a longer OS than placebo (53.3 months versus 36.5; HR = 0.66; *p* < 0.0001).

#### 2.3.3. Renal and Cardiac Toxicity

The mechanisms underlying hypertension caused by abiraterone are well known. The drug is able to inhibit the extra-gonadic production of testosterone by inhibiting the enzyme activity of steroid 17alpha-monooxygenase, which is a member of the cytochrome p450 family that catalyzes the 17alpha-hydroxylation of the steroid intermediates involved in testosterone synthesis, or the direct inhibition of androgen receptor activity [41]. CYP17 is the key enzyme inhibited by abiraterone, which bears components of 17alpha-hydroxylase and 17, 20-lyase and mediates androgen and cortisol synthesis. As a result of inhibiting CYP17, the level of aldosterone is elevated, and the levels of cortisol and dihydrotestosterone are suppressed. The inhibition of CYP17 can increase the concentration of mineral corticoid with resultant water retention, hypertension and the possible development of heart failure. 

With regards to cardiovascular toxicity, in the COU-AA 301 trial, 77 (10%) patients developed hypertension of all grades, including 10 (1%) cases of grade 3; 106 (13%) patients had cardiac disorders of all grades, including 26 (3%) of grade 3 and seven (1%) of grade 4. In the final analysis of COU-AA 301, hypertension of all grades was observed in 88 (11%) patients, 10 (1%) of grade 3. Finally, in the group of patients treated with abiraterone acetate, 126 (16%) patient had cardiac adverse events of all grades, 32 (4%) of grade 3 and nine (1%) of grade 4 [42]. A post hoc analysis of this trial assessed the efficacy and safety of abiraterone in elderly (≥75 years) and younger (<75 years) patients [43]. A slightly larger proportion of elderly patients taking abiraterone had cardiac adverse events, such as atrial fibrillation and tachycardia (5% versus 1% and 5% versus 2%, respectively). When adjusting for the duration of exposure, treatment-emergent atrial fibrillation and tachycardia in elderly patients were higher in the experimental arm than the control one (8% versus 2% and 6% versus 3%, respectively). The rates were similar in the younger patient groups (4% versus 4% and 5% versus 4 %, respectively). With regards to hypertension, in elderly patients, all-grade hypertension was evident in 20 (9%) and seven (6%) patients in the experimental and placebo arm, being of grade 3 in three (1%) and 0 patients in the experimental and placebo arm, respectively. In younger patients, all-grade hypertension was evident in 56 (10%) and 20 (7%) patients in the experimental and placebo arm, being of grade 3 in seven (1%) and one (<1%) of patients in the experimental and placebo arm, respectively.

In the COU-AA 302, 118 (22%) patients developed hypertension of every grade, including 21 (4%) of grade 3–4 and 102 (19%) developed cardiac disorder of all grade, 31 (6%) of grade 3–4. In particular, atrial fibrillation resulted the most common heart disease, being reported in 22 (4%) patients, seven (1%) of grade 3–4. In the final OS analysis, it was reported that 104 (19%) patients developed hypertension of grade 1–2, 25 (5%) of grade 3; 81 (15%) cases had a cardiac disorders of grade 1–2, 35 (6%) of grade 3, six (1%) of grade 4 and four (<1%) of grade 5 [44]. Grade 1–2 atrial fibrillation was reported in 20 (4%) men; eight (1%) of grade 3, two (<1%) of grade 4 and one (<1%) of grade 5. A post hoc analysis of this trial assessed the efficacy and safety of abiraterone in elderly (≥75 years) and younger (<75 years) patients [45]. The incidence of hypertension and grade 3–4 cardiac disorders was higher during abiraterone in both elderly and younger patients (16 [9%] versus 8 [5%] in the experimental and placebo arm, respectively). In particular, in elderly patients, all grade hypertension was evident in 40 (22%) and 28 (17%) patients in the experimental and placebo arm, being of grade 3–4 in eight (4.4%) and nine (6%) patients in the experimental and placebo arm, respectively. In younger patients, all-grade hypertension was evident in 78 (21.7%) and 45 (12%) patients in the experimental and placebo arm, being of grade 3–4 in 15 (4.2%) and eight (2.1%) patients in the experimental and placebo arm, respectively. With regards to cardiac disorders, in elderly patients, they were evident in 49 (27%) and 43 (26.2%) patients in the experimental and placebo arm, being of grade 3–4 in 16 (8.8%) and eight (5%) patients in the experimental and placebo group, respectively. In younger patients, cardiac adverse events were evident in 64 (17.8%) and 52 (13.8%) patients in the experimental and placebo arm, being of grade 3–4 in 20 (5.6%) and 11 (2.9%) patients in the experimental and placebo group, respectively.

In the STAMPEDE trial, cardiovascular disorders occurred in 92 (10%) patients, hypertension in 44 (5%), myocardial infarction in 10 (1%) and cardiac dysrhythmia in 14 (1%). 

In the LATITUDE trial, hypertension occurred in 229 (37%) patients, with grade 3 events in 125 (20%) cases. Cardiac disorders occurred in 74 (12%) patients, with grade 3 events in 15 (3%), grade 4 in five (1%) patients and grade 5 in 10 (2%). Atrial fibrillation was described in eight (1%) patients, with grade 3 events in two (<1%) cases.

A meta-analysis by Iacovelli et al. has investigated the cardiac toxicity and hypertension related to abiraterone and enzalutamide treatment in patients with PC [46]. The analysis includes 8600 patients and it has described a 36% increasing risk of cardiac events for new hormonal drugs versus ADT. Interestingly, when studies performed in patients with HSPC were compared with those performed in patients with CRPC, men treated with abiraterone with CRPC have significant major incidence of high-grade cardiac toxicity events compared with patients with HSPC, but no increase of all grades cardiac toxicity was found. This was probably related to the longer duration of ADT in patients with CRPC. Another meta-analysis by Moreira et al. evaluated the cardiovascular side effects of abiraterone plus prednisone versus placebo plus prednisone for the treatment of PC [47]. In the experimental arm, a 7% increase of high-grade cardiac disorders was described in comparison with the control arm. 

There are no clinical studies concerning a possible nephrotoxic effect determined by abiraterone acetate. In the COU-AA 301 trial, it was only reported an incidence of 8% (65 patients) for hematuria of all grades, including 1% (11 patients) of grade 3. The systemic exposure to abiraterone after a single oral dose was not increased in patients with terminal nephropathy undergoing dialysis, for which no dose reductions were necessary [48]. For this reason, in patients undergoing hemodialysis, no dose adjustments are indicated.

### 2.4. Enzalutamide

#### 2.4.1. Mechanism of Action, Metabolism and Clinical Use

Enzalutamide is a non-steroidal AR inhibitor that affects the AR pathway in different ways: (1) it binds AR with a greater affinity than bicalutamide; (2) it reduces the efficiency of AR nuclear translocation; (3) it impairs the recruitment of co-activators [49]. Enzalutamide is mainly metabolized by CYP2C8, and, to a lesser extent, by CYP3A4/5. The two main metabolites are the active N-desmethyl enzalutamide and the inactive carboxylic acid metabolite [50,51]. The highest elimination rate of the drug is renal (about 70% of total dose) [52]. Several interactions with drugs metabolized by CYP2C and CYP3A4/5 have been described. A recent study described possible interactions between co-medications and enzalutamide treatment in mCRPC [53]: carbamezepin is a strong CYP3A4 inducer and it can determine a reduction of enzalutamide exposure; moreover, an interaction between enzalutamide and pump proton inhibitors (PPI) is described resulting in a reduction of PPI biodisponibility. In addition, patients treated with aspirin or anticoagulant drugs are reported to have a greater risk of gastrointestinal bleeding. In consideration of the great capacity of induction of CYP3A4 by enzalutamide, a considerable blood level reduction of dexamethasone, nifedipine and fentanyl has been described. Enzalutamide was initially approved by the FDA in 2012 and by EMA in 2015 as a treatment for mCRPC previously treated with docetaxel. Subsequently, extensions of this indication were performed, so, now, according to the last FDA indications in 2019 and EMA indications in 2018, the treatment is approved for the following patients: (1) high-risk men with nmCRPR; (2) asymptomatic or mildly symptomatic patients with mCRPC after the failure of ADT and for whom chemotherapy is not still clinically indicated; (3) patients with mCRPC, whose disease has progressed during or after treatment with docetaxel [54]. 

#### 2.4.2. Clinical Trials

The AFFIRM trial is a phase III study in which 1199 patients with mCRPC previously treated with chemotherapy were randomized in a 2:1 ratio to receive enzalutamide versus placebo [55]. The primary objective of the trial was OS and it was met since patients in the experimental arm had a better survival than patients in the placebo group (18.4 versus 13.6 months in the experimental and placebo arm, respectively; HR = 0.63; *p* < 0.001).

The PREVAIL study is a phase III trial that randomized in a 1:1 ratio 1717 men with chemotherapy-naive mCRPC to receive enzalutamide versus placebo [56]. The co-primary objectives of the trial were the radiographic PFS and OS and they both favored the experimental arm since the rate of radiographic PFS at 12 months was 65% versus 14% in the placebo arm (HR = 0.19; *p* < 0.001) and the median OS was 32.4 versus 30.2 months in the experimental and placebo group, respectively (HR = 0.71; *p* < 0.001). 

Another phase III study, the PROSPER trial, randomized in a 2:1 ratio 1401 patients with nmCRPC to receive enzalutamide versus placebo. The primary objective of the trial was metastasis-free survival [57]. The results show a metastasis-free survival more than doubled for patients receiving enzalutamide (36.6 months versus 14.7 months in the experimental versus placebo group, respectively; HR = 0.29; *p* < 0.001). Moreover, a total of 219/933 patients (23%) in the enzalutamide group had metastasis or had died, as compared with 228/468 (49%) in the placebo group. 

#### 2.4.3. Renal and Cardiac Toxicity

As regarding cardiac toxicities, in the AFFIRM study they occurred in 49 (6%) patients, with seven (1%) of grade 3–4. Hypertension was observed in 52 (6.6%) men and myocardial infarction in two (<1%) cases of severe grade.

In the PREVAIL trial, hypertension was the most common cardiovascular events occurring in 117 (13%) patients, 59 (7%) of grade 3–4. Cardiac events were observed in 88 (10%) cases, 24 (3%) of grade 3–4. Atrial fibrillation occurred in 16 (2%) cases in the enzalutamide group including 3 (<1%) of severe grade. Acute coronary syndrome was rarer and in seven (1%) patients of grade 3–4.

In the PROSPER trial, 111 (12%) patients developed hypertension of all grade and 43 (5%) of grade 3–4; 48 (5%) patients had adverse cardiovascular events, 34 (4%) of grade 3–4. In a minority of cases, the cardiovascular event leads to death and, in particular, six (<1%) patients had an acute myocardial infarction and one (<1%) patient a cardiac failure, with ventricular arrhythmia and subsequent cardiorespiratory arrest. 

In the aforementioned meta-analysis by Iacovelli et al., a greater risk of hypertension was reported, particularly in patients treated with enzalutamide with an incidence of all- and high-grade of 10.5% and 4.8%, respectively. The incidence of all- and high-grade cardiac toxicity by enzalutamide was 8.6% and 2.5%, respectively; these were not significantly increased compared to the placebo. However, in the meta-analysis by Moreira et al., enzalutamide has not been associated with an increased risk of cardiovascular toxicity.

As regards renal toxicity, few data are available. In the PROSPER trial, 62 (7%) patients reported hematuria in the enzalutamide group, including 16 (2%) of grade 3–4 and 20 (2%) had urinary retention, with four (<1%) of grade ≥3. In the PREVAIL trial, acute renal failure was described in 32 (4%) patients in the enzalutamide arm, 12 (1%) of grade 3–4. 

Considering that the elimination of enzalutamide is mainly renal, it has been reported that there are no differences in drug clearance in patients with mild-moderate renal impairment, even though patients who underwent hemodialysis were not well studied.

## 3. Chemotherapy 

### 3.1. Cabazitaxel

#### 3.1.1. Mechanism of Action, Metabolism and Clinical Use

Cabazitaxel is a novel tubulin-binding taxane drug, which is extensively metabolized by the liver (>95%), mainly by the CYP3A4 isoenzyme [58,59]. After a 1-h intravenous infusion at the standard dose of 25 mg/m^2^, about 80% of the drug was eliminated in 2 weeks. Cabazitaxel is mainly excreted in the feces in the form of metabolites (76% of the dose), while renal excretion is less than 4% of the total dose. The concomitant administration of strong CYP3A4 inhibitors, such as ketoconazole, itraconazole and clarithromycin, may increase the cabazitaxel concentrations [60]. While the concomitant administration of strong CYP3A inducers, such as phenytoin, carbamazepine and rifampin may reduce cabazitaxel bloods levels. A recent pharmacokinetics cross-over study in patients with mCRPC evaluated the concomitant administration of cabazitaxel and enzalutamide and it found a significant and potential clinically important reduction (22%) in cabazitaxel exposure when combined with enzalutamide [61]. The drug was approved by the FDA in 2010 and by the EMA in 2012, in combination with prednisone or prednisolone, for the treatment of patients with mCRPC previously treated with a regimen containing docetaxel [62].

#### 3.1.2. Clinical Trials

In the phase III TROPIC trial, 765 patients affected by mCRPC metastatic previously treated with docetaxel were randomized 1:1 to receive cabazitaxel 25 mg/m^2^ versus mitoxantrone [63]. The primary objective of the study was OS and the results favored the experimental arm with a median OS of 15.1 months in the cabazitaxel group versus 12.7 months in the mitoxantrone one (HR = 0.70; *p* < 0.0001). 

In the phase III PROSELICA trial, 1200 patients with mCRPC were randomized 1:1 to receive cabazitaxel 20 mg/m^2^ versus cabazitaxel 25 mg/m^2^ [58]. The primary objective of the study was to demonstrate the non-inferiority of cabazitaxel 20 mg/m^2^ versus cabazitaxel 25 mg/m^2^ in post-docetaxel treatment. The results show a median OS of 13.4 months for the cabazitaxel 20 mg/m^2^ group and 14.5 months for the cabazitaxel 25 mg/m^2^ one (HR = 1.024; *p* < 0.001), so the primary objective of non-inferiority was met. Authors concluded that cabazitaxel 25 mg/m^2^ was an appropriate starting dose, and, if subsequent dose reduction become necessary due to adverse events, cabazitaxel could be reduced to 20 mg/m^2^ without detrimental effects on patient outcomes.

The CARD trial is a phase III study in which 255 patients with mCRPC previously treated with docetaxel and an androgen-signaling targeted inhibitor, such as abiraterone or enzalutamide, were randomized 1:1 to receive cabazitaxel plus prednisone (experimental arm) versus an androgen-signaling targeted inhibitor (abiraterone or enzalutamide) not previously used (control arm) [64]. The primary objective of the study was the imaging-based PFS. The results show a better outcome for the cabazitaxel arm with an imaging-based PFS of 73.6% in the chemotherapy group, as compared 80.2% in the control group (HR = 0.54; *p* < 0.001).

#### 3.1.3. Renal and Cardiac Toxicity

Few data regarding the cardiovascular and renal toxicity of cabazitaxel are reported. 

In the TROPIC trial, five (1%) patients had cardiac adverse events and three (1%) patients developed renal failure. A total of 62 (17%) patients complained of hematuria, including 7 (2%) of grade ≥3.

No cardiac toxicity was reported in the PROSELICA trial; a total of 82 (14.1%) men developed haematuria of every grade with 11 (1.9%) cases of grade ≥3.

In the CARD trial, hypertension was described in five (4%) patients in the cabazitaxel arm, and three (2.4%) of grade ≥3. Hematuria was described in 19 (15.1%) cases with only one (0.8%) case of grade ≥3. Renal disorders occurred in eight (6.3%) patients, with four (3.2%) cases of grade ≥3. In the control group, hypertension was recorded in 10 (8%) patients, and three (2.4%) of grade ≥3; 10 (8.1%) patients developed all-grade cardiac disorders, including six (4.8%) of grade ≥3. Hematuria was described in seven (5.6%) patients (all grade), and two (1.6%) of grade ≥3. Renal disorders involved 14 (11.3%) cases, and 10 (8.1%) of grade ≥3.

In the German compassionate use program of cabazitaxel, grade 3–4 myocardial infarction and pulmonary embolism affected one (0.9%) man [65]. Similar results were reported by the UK expanded access program in which one (0.9%) man developed a cardiac toxicity [66]. In the Dutch compassionate use program, myocardial infarction was reported in one (2%) patient (grade 3–4) [67].

Altered renal function is not likely to affect the pharmacokinetics of the drug [68]. Clinical trials showed that cabazitaxel clearance was similar for patients with kidney disease and for patients without renal disorders; therefore, cabazitaxel dose modification is not required for patients with moderate or severe renal dysfunction [69,70,71]. Moreover, full-dose cabazitaxel (25 mg/m^2^) can be safely administered every 3 weeks to patients with mild-to-severe renal impairment [72].

## 4. Radioisotope Therapy

### 4.1. Radium-223

#### 4.1.1. Mechanism of Action, Metabolism and Clinical Use

Radium-223 dichloride is a novel target radioisotope that binds mineral hydroxyapatite of newly formed bone stroma in the bone portions of increased metabolic activity, such as bone metastasis [73]. The drug determines the emission of high-energy alpha-particle radiation of short range (<100 mm; <10 cell diameters) with the consequent breaking of the double-helium DNA with a cytotoxic effect in the area concerned, limiting the damage on adjacent tissues, in particular the bone marrow. With regards to drug metabolism, after 24 h from the injection of radium-223, the quantity of drug inside the body is closed to 85% [74]. At the bone level, the initial uptake is about 52% within two hours (range 41–57%), with the maximum activity of the drug occurring within the 2 h themselves. Within 24 h, the drug passes through the intestinal barrier. Urinary excretion is minimal (at 48 h it reaches 2%), whereas faecal excretion at 72 h from distribution is about 64%. No drug interactions with radium 223 have been reported [75]. Radium-223 was approved by the FDA and EMA in 2013 for the treatment of mCRPC with symptomatic bone metastases, in the absence of visceral secondarysms [76].

#### 4.1.2. Clinical Trials

In a phase III study, the ALSYMPCA trial, 921 patients affected by mCRPC were randomized in a 2:1 ratio to receive radium-223 versus placebo [77]. The primary objective of the study was OS and it was met since the experimental arm significantly improved survival compared to the control group (14.0 versus 11.2 months for the experimental and placebo arm, respectively; HR = 0.70; *p* = 0.002).

#### 4.1.3. Renal and Cardiac Toxicity

In the ALSYMPCA trial, no cardiovascular adverse events were reported. Hematuria affected 30 (5%) patients in the experimental arm including seven (1%) cases of grade 3 [78], and urinary retention occurred in 25 (4%) patients, with nine (2%) of grade 3–4. Considering the almost complete faecal metabolism of the drug, with minimal elimination through the urine, no dose reductions in patients with renal insufficiency are prescribed.

## 5. Prevention, Follow-Up and Treatment of Adverse Events

The general principles to prevent and manage cardiac and renal adverse events of PC patients treated with new drugs are: (1) to assess the need of the drug and make decisions regarding its use in a joint decision-making process with both patients, cardiologist and nephrologists; (2) identify patients at high risk of complications; (3) proactively prevent, reduce and address the problems that may arise from these treatments.

The occurrence of serious adverse events related to the administration of treatments for PC is significantly related to a worse performance status. The likelihood of treatment effectiveness or success in unfit patients will be influenced by these side effects which prevent the administration of full doses and cause delays or complications that, in a worst-case scenario, may prove to be fatal. The International Society of Geriatric Oncology (SIOG) working group has established that elderly patients with PC should be managed according to their health status, which is mainly determined by their comorbidities, not by chronological age [79,80].

Cardiotoxicity represents a fundamental issue in oncology clinical practice, since it correlates with poor outcome in cancer patients [81]. Basal assessment should consist of a careful medical history, physical examination, ECG, blood tests and subsequent instrumental tests. Given the significant impact of cardiovascular side effects in some subsets of patients, it is appropriated to undertake interventions that can reduce this risk and indirectly increase cancer-specific survival. Therefore, it is necessary to intervene as early as possible on editable cardiovascular risk factors [82]. 

An early recognition of renal impairment or even its prevention represents an important aspect to minimize the risk of renal injury. This can be accomplished by a routine use of glomerular filtration rate (eGFR) estimation rather than serum creatinine value alone (which may be in the normal range in older patients or those with muscle wasting) [83,84]. Moreover, a urine protein assessment should be performed to identify patients with pre-existing renal impairment, or a close monitoring of urine output to detect an acute reduction in renal function. These scenarios may dictate a need to reduce drug doses, to avoid nephrotoxic agents and to administer intravenous fluids to correct volume depletion [85]. 

The multidisciplinary management of cardiac and renal toxicities by oncologist, cardiologist and nephrologist is a guarantee for an optimal continuation of specific cancer treatment. In addition, it is necessary to have a good knowledge of the molecular mechanisms of actions, pharmacokinetics and potential interactions of anti-cancer treatments with concomitant medications taken by the patient. The appropriate management should include a better detection of high-risk patients and an immediate treatment by the specialist when an adverse event appears. This fact should be emphasized, even in patients younger than 65 years old. 

## 6. Conclusions

PC is one of the most common forms of cancer in the world. Age and comorbidities are independent prognostic factors for many cancers with an impact on the treatment choice [86]. This is particularly true for PC because it is primarily a disease of elderly men. Several drugs have been recently developed for high-risk or metastatic disease with an improvement in patient outcomes (Table 1). In particular, different hormonal drugs have been approved, determining a treatment alternative to chemotherapy, particularly in frail patients with multiple comorbidities. 

This review analyzed and described the incidence of cardiovascular and renal toxicity in patients treated with new drugs, as summarized in Table 2 and Table 3. It emerged that the main side effect is hypertension, in particular derived from treatment with new hormonal agents, abiraterone and enzalutamide, or new non-steroidal androgen receptor drugs, in particular apalutamide. Moreover, the incidence of cardiac disorders can be particularly evident for abiraterone and enzalutamide followed by apalutamide and darolutamide. Atrial fibrillation is rarer and mainly described during abiraterone and enzalutamide therapy. Renal adverse events are confirmed to be even rarer and mainly affecting patients treated with cabazitaxel.

However, it is important to underline that patients enrolled in clinical trials generally had adequate organ function and those with chronic or multiple concomitant diseases are excluded. As a result of these selection criteria, the incidence of cardiovascular and renal events is expected to be higher in an unselected population.

Moreover, increasing age and pre-existing relevant medical conditions can increase the probability to develop a new or worsened comorbid disease during anti-cancer treatments. 

For that reason, patients should be investigated for pre-existing risk factors in order to optimize those that are modifiable, even if consensus recommendations for the identification of a population most at risk of toxic events are currently lacking. For those patients with baseline organ impairments, a multidisciplinary approach is strongly recommended for an early identification of new treatment-related cardiovascular and renal side effects.

## Figures and Tables

**Table 1:** Clinical trial involving new drugs for the treatment of advance prostate cancer.

Author Year [Ref]	Trial	Study Design	N Patients	Treatment Line	Drug	Primary Endpoints
Sandler, H.M. et al. 2016 [25]	ATLAS	Phase III Placebo controlled Double-blind Randomized 1:1	1503	High risk, localized or locally advanced HSPC	APA *versus* BICA or APA *versus* plb	Metastasis-free survival
Smith, M.D. et al. 2018 [24]	SPARTAN	Phase III Placebo controlled Double blind Randomized 2:1	1207	Non metastatic CRPC	APA *versus* plb	Metastasis-free survival
Chi, K.N. et al. 2019 [26]	TITAN	Phase III Placebo controlled Double blind Randomized 1:1	1052	mHSPC	APA *versus* plb	Radiographic-free survival OS
Fizazi, K. et al. 2014 [32]	ARADES	Phase I: Non randomized Dose-escalation Phase II: Randomized 1:1	136	mCRPC	Phase I: DARO 200 mg–1800 mg Phase II: DARO 200 mg–400 mg–1400 mg	Phase I: Safety and tolerability Phase II: PSA response at 12 week
Smith, M. R. et al. 2018 [34]	ARASENS	Phase III Placebo-controlled Double-blind Randomized 1:1	1300	mHSPC	DARO + DCT + ADT *versus* plb + DCT + ADT	OS
Fizazi, K. et al. 2018 [33]	ARAMIS	Phase III Placebo controlled Double-blind Randomized 2:1	1509	Non metastatic CRPC	DARO + ADT *versus* plb + ADT	Metastasis-free survival
De Bono, J.S. et al. 2011 [35]	COU-AA-301	Phase III Placebo controlled Double-blind Randomized 2:1	1195	mCRPC post- DCT	ABI *versus* plb	OS
Ryan, C.J. et al. 2013 [38]	COU-AA-302	Phase III Placebo controlled Double-blind Randomized 1:1	1088	mCRPC pre-CT	ABI *versus* plb	Radiographic progression-free survival OS
James, N. D. et al. 2017 [39]	STAMPEDE	Phase III Double-blind Randomized 1:1	1917	Metastatic and non-metastatic, high-risk, treatment-naive	ADT *versus* ADT + ABI	OS
Fizazi, K. et al. 2019 [40]	LATITUDE	Phase III Placebo controlled Double-blind Randomized 1:1	1199	mHSPC	ADT + ABI *versus* ADT + plb	Radiographic- free survival OS
Sher, H.I. et al. 2012 [55]	AFFIRM	Phase III Placebo controlled Double-blind Randomized 2:1	1199	mCRPC post-CT	ENZA *versus* plb	OS
Beer, T.M. et al. 2014 [56]	PREVAIL	Phase III Placebo controlled Double-blind Randomized 1:1	1717	mCRPC pre-CT	ENZA *versus* plb	Radiographic progression- free survival OS
Hussain, M. et al. 2018 [57]	PROSPER	Phase III Placebo controlled Double-blind Randomized 2:1	1401	Non metastatic CRPC	ENZA *versus* plb	Metastasis-free survival
De Bono, J.S. et al. 2010 [63]	TROPIC	Phase III Double-blind Randomized 1:1	765	mCRPC post-DCT	MITO *versus* CABA	OS
Eisenberger, M. et al. 2017 [58]	PROSELICA	Phase III Double-blind Randomized 1:1	1200	mCRPC post-DCT	CABA 20 mg/m^2^ *versus* CABA 25 mg/m^2^	Non-inferiority of CABA 20 mg/m^2^
De Wit R. et al. 2019 [64]	CARD	Phase IV Open label Randomized 1:1	255	mCRPC post-DCT, ABI, ENZA	CABA *versus* ABI or ENZA	Imaging – based progression-free survival
Parker C. et al. 2013 [77]	ALSYMPCA	Phase III Placebo controlled Double-blind Randomized 2:1	921	mCRPC	Radium 223 *versus* plb	OS

Abbreviations: ABI: abiraterone; ADT: androgen deprivation therapy; APA: apalutamide; BICA: bicalutamide; CABA: cabazitaxel; CT: chemotherapy; DARO: darolutamide; DCT: docetaxel; ENZA: enzalutamide; mCRPC: metastatic castration-resistant prostate cancer; mHSPC: metastatic hormone-sensitive prostate cancer; MITO: mitoxantrone; overall survival: OS; plb: placebo.

**Table 2:** Incidence of cardiovascular adverse events of new drugs for advance prostate cancer treatment.

Disease Setting	Trial	Drugs (Experimental Arm/Control Arm)	Hypertension			Atrial Fibrillation			Cardiac Disorders *		
			ALL GRADE (%)	GRADE 3–4 (%)	GRADE 5 (%)	ALL GRADE (%)	GRADE 3–4 (%)	GRADE 5 (%)	ALL GRADE (%)	GRADE 3–4 (%)	Grade 5 (%)
mHSPC	TITAN	APA/plb	17.7/15.6	8.4/9.1	NR	0/0	0/0	NR	4.4/<1	0/0	<1/<1
	ARASENS	DARO + DCT + ADT/plb + DCT + ADT	0/0	0/0	NR	0/0	0/0	NR	0/0	0/0	NR
	LATITUDE	ADT + ABI/ADT + plb	37/22	20/10	NR	1/<1	<1/<1	NR	12/8	4/1	2/1
Non metastatic CRPC	SPARTAN	APA/plb	24.8/19.8	14.3/11.8	NR	<1/0	0/0	NR	<1/0	0/0	<1/0
	ARAMIS	DARO + ADT/plb + ADT	6.6/5.2	3.1/2.2	0/<1	0/0	0/0	NR	5.1/3.4	2.2/<1	1/1.2
	PROSPER	ENZA/plb	12/5	5/2	NR	0/0	0/0	NR	5/3	4/2	1/<1
Metastatic or not, treatment naive	STAMPEDE	ADT + ABI/ADT	1/5	NR	NR	0/0	NR	NR	2/1	NR	2/0
mCRPC pre-CT	COU-AA-302	ABI/plb	24/14	5/3	0/0	5/5	<2/0	<1/0	22/18	7/3.5	<1/<1
	PREVAIL	ENZA/plb	13/4	7/2	NR	2/1	<1/1	NR	10/8	3/2	NR
mCRPC	ARADES	DARO	7.2	<1	NR	0	0	NR	0	0	NR
	COU-AA-301	ABI/plb	11/8	1/<1	NR	0/0	0/0	NR	16/12	5/2	NR
	AFFIRM	ENZA/plb	6.6/3.3	NR	NR	0/0	NR	NR	6/8	1/2	NR
	TROPIC	MITO/CABA	0/0	0/0	NR	0/0	0/0	NR	0/1	0/0	0/1
	PROSELICA	CABA 20 mg-m^2^/CABA 25 mg-m^2^	0/0	0/0	NR	0/0	0/0	NR	0/0	0/0	NR
	CARD	CABA/ABI or ENZA	4/8	2.4/ 2.4	0/<1	0/0	0/0	NR	6.3/8.1	<1/4.8	0/1
	ALSYMPCA	Radium 223/plb	0/0	0/0	NR	0/0	0/0	NR	0/0	0/0	NR

Abbreviations: ABI: abiraterone; ADT: androgen deprivation therapy; APA: apalutamide; CABA: cabazitaxel; CT: chemotherapy; DARO: darolutamide; DCT: docetaxel; ENZA: enzalutamide; mCRPC: metastatic castration-resistant prostate cancer; mHSPC: metastatic hormone-sensitive prostate cancer; MITO: mitoxantrone; NR: not reported; plb: placebo; * Cardiac disorders include ischemic heart disease, myocardial infarction, arrhythmias, and cardiac failure.

**Table 3:** Incidence of renal adverse events of new drugs for advance prostate cancer treatment.

Disease Setting	Trial	Drugs (Experimental Arm/Control Arm)	Hematuria			Renal Failure		
			ALL GRADE (%)	GRADE 3–4 (%)	GRADE 5 (%)	ALL GRADE (%)	GRADE 3–4 (%)	GRADE 5 (%)
mHSPC	TITAN	APA/plb	0/0	0/0	NR	2.5/3.6 *	<1/1.9 *	<1/0 *
	ARASENS	DARO + DCT + ADT/plb + DCT + ADT	0/0	0/0	NR	0/0	0/0	NR
	LATITUDE	ADT + ABI/ADT + plb	0/0	0/0	NR	0/0	0/0	0/0
Non metastatic CRPC	SPARTAN	APA/plb	1.1/0	0/0	NR	<1/0	0/0	NR
	ARAMIS	DARO + ADT/plb + ADT	0/0	0/0	NR	3.5/6.5 *	1.6/2 *	NR
	PROSPER	ENZA/plb	7/8	2/3	NR	2/6 *	<1/1 *	NR
Metastatic or not, treatment naive	STAMPEDE	ADT/ADT + ABI	0/0	0/0	0/0	0/0	0/0	NR
mCRPC pre-CT	COU-AA-302	ABI/plb	0/0	0/0	0/0	0/0	0/0	0/0
	PREVAIL	ENZA/plb	0/0	0/0	NR	4/5	1/1	NR
mCRPC	ARADES	DARO	0/0	0/0	NR	0/0	0/0	NR
	COU-AA-301	ABI/plb	8/8	1/2	NR	0/0	0/0	NR
	AFFIRM	ENZA/plb	0/0	0/0	NR	0/0	0/0	NR
	TROPIC	MITO/CABA	17/4	2/1	NR	0/1	0/0	0/1
	PROSELICA	CABA 20 mg-m^2^/CABA 25 mg-m^2^	14.1/20.8	1.9/4.2	NR	0/0	0/0	NR
	CARD	CABA/ABI or ENZA	15.1/5.6	<1/1.6	NR	6.3/11.3	3.2/8.1	0/1
	ALSYMPCA	Radium 223/plb	5/5	1/1	0/0	4/6 *	2/2 *	0/0

Abbreviations: ABI: abiraterone; ADT: androgen deprivation therapy; APA: apalutamide; CABA: cabazitaxel; CT: chemotherapy; DARO: darolutamide; DCT: docetaxel; ENZA: enzalutamide; mCRPC: metastatic castration-resistant prostate cancer; mHSPC: metastatic hormone-sensitive prostate cancer; MITO: mitoxantrone; NR: not reported; plb: placebo. * Urinary retention.
